# Recombinant Soybean Lipoxygenase 2 (GmLOX2) Acts Primarily as a ω6(*S*)-Lipoxygenase

**DOI:** 10.3390/cimb45080396

**Published:** 2023-07-28

**Authors:** Elena O. Smirnova, Alevtina M. Egorova, Natalia V. Lantsova, Ivan R. Chechetkin, Yana Y. Toporkova, Alexander N. Grechkin

**Affiliations:** Kazan Institute of Biochemistry and Biophysics, FRC Kazan Scientific Center of RAS, P.O. Box 261, 420111 Kazan, Russia; egorova@kibb.knc.ru (A.M.E.); natamed2@yandex.ru (N.V.L.); adequatus@yandex.ru (I.R.C.);

**Keywords:** soybean, lipoxygenase-2, fatty acid oxidation, substrate specificity, regio- and stereospecificity

## Abstract

The lipoxygenase (LOX) cascade is a source of bioactive oxylipins that play a regulatory role in plants, animals, and fungi. Soybean (*Glycine max* (L.) Merr.) LOXs are the classical models for LOX research. Progress in genomics has uncovered a large diversity of GmLOX isoenzymes. Most of them await biochemical investigations. The catalytic properties of recombinant soybean LOX2 (GmLOX2) are described in the present work. The GmLOX2 gene has been cloned before, but only for nucleotide sequencing, while the recombinant protein was not prepared and studied. In the present work, the recombinant GmLOX2 behavior towards linoleic, α-linolenic, eicosatetraenoic (20:4), eicosapentaenoic (20:5), and hexadecatrienoic (16:3) acids was examined. Linoleic acid was a preferred substrate. Oxidation of linoleic acid afforded 94% optically pure (13*S*)-hydroperoxide and 6% racemic 9-hydroperoxide. GmLOX2 was less active on other substrates but possessed an even higher degree of regio- and stereospecificity. For example, it converted α-linolenic acid into (13*S*)-hydroperoxide at about 98% yield. GmLOX2 showed similar specificity towards other substrates, producing (15*S*)-hydroperoxides (with 20:4 and 20:5) or (11*S*)-hydroperoxide (with 16:3). Thus, the obtained data demonstrate that soybean GmLOX2 is a specific (13*S*)-LOX. Overall, the catalytic properties of GmLOX2 are quite similar to those of GmLOX1, but pH is optimum.

## 1. Introduction

Lipoxygenases (LOXs, EC 1.13.11.12) are non-haem iron-containing oxidoreductases that catalyze the dioxygenation of polyenoic fatty acids to produce the corresponding conjugated hydroperoxy derivatives [1,2,3,4]. Most LOXs exhibit high regio- and stereospecificity. For instance, plant LOXs transform linoleate and linolenate into either (13*S*)- or (9*S*)-hydroperoxides [5]. This oxidation is the initial stage of the plant LOX cascade, producing the numerous oxylipins that play signaling, growth-regulating and defensive roles in plants [6,7]. Separate plant genomes encode multiple LOX isoforms that differ both in the amino acid sequences and the catalytic properties [8]. Different LOX isoenzymes possess distinct expression specificities, depending on tissue, subcellular compartments, stage of ontogenesis, and numerous other factors [9]. One can propose that the significance of separate isoenzymes is still underestimated. The great majority of LOX isoenzymes await their cloning and characterization.

Currently, the genomic studies on soybean (*Glycine max* (L.) Merr.) have uncovered at least 37 expressed LOX genes and 11 pseudogenes. Three genes from soybean seeds (encoding L-1 (GmLOX1), L-2 (GmLOX2 [10]), and L-3 (GmLOX3)) and five genes from vegetative tissues (encoding VLXA, B, C, D, and E enzymes) [8,9] have been cloned. The corresponding recombinant proteins have been characterized except for GmLOX2 and GmLOX3 [8,9,10,11,12,13,14,15]. The biochemical properties of GmLOX2, purified from soybeans, were studied in the early literature [16,17,18,19,20,21,22]. According to these data [16,17,18,19,20,21,22], GmLOX2 has dual 13/9-LOX specificity. The yield of 9-hydroperoxide reported by different authors varied between 20 and 90% [16,17,18]. Thus, the available data on the regio- and stereospecificity of GmLOX2 are contradictory and need clarification. The present work is concerned with the preparation of recombinant GmLOX2 and studies of the specificity of its catalysis.

## 2. Materials and Methods

### 2.1. Materials

Linoleic, α-linolenic, hexadecatrienoic, arachidonic, and eicosapentaenoic acids, and isopropyl β-D-thiogalactoside were purchased from Sigma. NaBH_4_ and silylating reagents were purchased from Fluka (Buchs, Switzerland). The plasmid pUC3C76 carrying the full-length soybean L-2 (*GmLOX2*) cDNA (GenBank GeneID: 547774) was generously provided by Prof. D. Shibata and Dr. T. Nagaya. Oligonucleotides were obtained from Evrogen (Moscow, Russia).

### 2.2. Bioinformatics Studies

The search for soybean LOX genes was carried out in the NCBI database. Primer construction was performed using the Vector NTI Advance 11.5 program (Invitrogen, Madison, WI, USA). The BLAST analyses of the LOXs were performed using the protein NCBI BLAST tool. The multiple alignments of selected LOX amino acid sequences were made with Clustal Omega and MEGA5 software [23]. The evolutionary history was inferred by using the Maximum Likelihood method based on the JTT matrix-based model [24]. The tree with the highest log likelihood (−29002.7382) is shown. The initial tree(s) for the heuristic search were obtained automatically by applying the Neighbor-Join and BioNJ algorithms to a matrix of pairwise distances estimated using a JTT model and then selecting the topology with the superior log likelihood value. The tree is drawn to scale, with branch lengths measured in the number of substitutions per site. The analysis involved 42 amino acid sequences. All positions containing gaps and missing data were eliminated. There were a total of 734 positions in the final dataset. Evolutionary analyses were conducted in MEGA5 [25]. The iTOL tool was used to visualize the phylogenetic model output. 

### 2.3. Cloning and Expression of Recombinant GmLOX2

The GmLOX2 encoding sequence was subcloned into the expression vector pET-32 Ek/LIC (Novagene, Madison, WI, USA) using the ligation-independent cloning method. The resulting construct was transformed into the *E. coli* host strain Rosetta-gami(DE3)pLysS B. Selected clones were controlled by DNA sequence analysis. A clone having the sequence published previously by Shibata et al. [10] (GenBank GeneID: 547774) was used for protein expression.

The recombinant GmLOX2 was obtained as follows. An overnight culture (10 mL) of bacteria was inoculated into 1 L of Luria-Bertani medium supplemented with mineral medium M9 (1:1, by volume). Bacteria were grown at 37 °C and 220 rpm to an OD_600_ of 0.6. Cultures were cooled to 18 °C, and isopropyl *β*-D-thiogalactoside was added to a final concentration of 0.1 mM. Induced cultures were incubated for 24 h at 18 °C with gentle shaking (110 rpm). Induction was controlled by the 12% SDS-PAGE analysis of crude cellular extracts. Cells were collected by centrifugation and stored at –85 °C until use. Thawed pellets were suspended in 10 mL of 50 mM phosphate buffer, pH 8.0, containing 100 mM NaCl and 0.3% polyoxyethylene (10) tridecyl ether (emulphogene).

### 2.4. Purification of the Recombinant GmLOX2

The recombinant protein-containing *E. coli* pellet was resuspended in BugBuster Protein Extraction Reagent according to the manufacturer’s recommendations. For the purification of the recombinant GmLOX2 from the cell lysate, anion-exchange chromatography was performed on a BabyBio Q 1 mL cartridge (Bio-Works, Uppsala, Sweden) and an NGC Discover 10 chromatographic system (Bio-Rad, Hercules, CA, USA). The protein was eluted with a linear gradient of NaCL (10–700 mM) in 50 mM Tris-HCl buffer (pH 8.0). The elution was monitored by absorption at 280 nm. The presence of GmLOX2 in the collected fractions was determined using 12.5% SDS-PAGE electrophoresis. Protein bands in gel were stained with Coomassie Brilliant Blue G-250. To confirm the presence of GmLOX2, the band of interest was cut from the gel and analyzed using HPLC-MS using a MicrOTOF-Q mass spectrometer (Bruker Daltonics, Bremen, Germany). The trypsinolysis and LC-MS analysis were conducted as described in [26]. The Mascot Deamon software version 2.2.6 (Matrix Science Inc., London, UK) was used for the MS/MS spectra search. The following searching parameters were applied: precursor ion mass tolerance of 0.1 Da, fragment ion mass tolerance of 0.1 Da, one missed cleavage, possible carbamidomethylation of cysteines, and oxidation of methionine modifications. Hits were considered reliable if at least two peptides were matched in the Mascot analysis with an ion score that indicated identity or extensive homology (*p* < 0.05). The analyzed protein was reliably identified as soybean GmLOX2, with a total score of 1150 and protein sequence coverage of 40%.

### 2.5. Studies of the Substrate Specificity and pH Optimum

The standard assay mixture (2 mL), containing 0.0032% Tween 20 and 140 μM linoleate, α-linolenate, arachidonate, hexadecatrieonate, and eicosapentaenoate in 50 mM sodium phosphate buffer (pH 6.5), was preliminary bubbled with oxygen for 3 min. The reaction was started by the addition of purified GmLOX2 preparation (0.028 U) and proceeded for 40 s at 23 °C. Enzymatic activity was determined by monitoring the increase in absorbance at 234 nm in a Spekol 1200 (Analytic Jena, Germany) UV/VIS spectrophotometer. The kinetic parameters were measured at the steady-state phase of the enzymatic reaction.

For analyses of pH optimum, linoleic acid was incubated with GmLOX2 in 50 mM sodium phosphate buffers with pH values of 6.2, 6.5, 6.6, 6.7, 6.8, 6.9, and 7.0, or 50 mM Tris/HCl buffers with pH values of 7.5 and 9.0.

### 2.6. Incubations of Free Fatty Acids with Recombinant GmLOX2

The recombinant GmLOX2 (0.14 U) was incubated with 1 mg of linoleate, α-linolenate, arachidonate, hexadecatrieonate, or eicosapentaenoate in 50 mM Tris/HCl buffer (pH 9.0) at 23 °C for 20 min under continuous oxygen bubbling and stirring. Incubations were terminated by the addition of glacial acetic acid to pH 5.0, followed by triple extraction with 10 mL of hexane/ethyl acetate (1:1, by volume). After solvent evaporation, the dry residue was dissolved in methanol and reduced with sodium borohydride. The reduced free fatty acids were methylated with diazomethane. The methyl esters were analyzed by NP-HPLC for the elucidation of GmLOX2 stereospecificity. Alternatively, the methyl esters were catalytically hydrogenated over PtO_2_ [27]. Hydrogenated samples were silylated with pyridine-hexamethyldisilazane-trimethylchlorosilane 2:1:2 (by volume) as described before [28]. The resulting Me/TMS derivatives were analyzed by GC-MS for the elucidation of GmLOX2 regiospecificity.

### 2.7. HPLC analysis of Products

The products were separated, at first, by NP-HPLC on two successively connected Separon SIX columns (5 µm; 3.2 × 150 mm; Tessek, Prague, Czech Republic) under isocratic conditions using the solvent mixture hexane/propan-2-ol/acetic acid 98.4:1.5:0.1 (by volume), at a flow rate of 0.4 mL/min. Peaks of separate products were collected and then subjected to CP-HPLC analysis on a Chiralcel OD-H column (5 µm; 4.6 × 250 mm; Daicel Chemical Industries, France) under isocratic conditions using the solvent mixture hexane/propan-2-ol 97:3 (by volume) at a flow rate of 0.4 mL/min. UV spectra of compounds being purified by HPLC were recorded on line using an RSD 2140 diode array detector and Wavescan software (LKB, Bromma, Sweden).

### 2.8. Methods of Spectral Analyses

Products (Me/TMS, after NaBH_4_ reduction and hydrogenation) of fatty acid conversions by the recombinant GmLOX2 were analyzed by GC-MS using a Shimadzu QP2020A mass spectrometer connected to a Shimadzu GC-2010 Plus gas chromatograph equipped with a Macherey-Nagel Optima-5-MS (5% phenyl, 95% methylpolysiloxane) fused capillary column (length, 30 m; ID, 0.25 mm; film thickness, 0.25 μm). Helium at a linear velocity of 30 cm/s was used as the carrier gas. Injections were made in split mode using an initial column temperature of 120 °C and an injector temperature of 230 °C. Then, the column temperature was raised at 10 °C/min until 240 °C. Electron impact ionization (70 eV) was used.

## 3. Results

### 3.1. Preparation of the Recombinant GmLOX2 and Studies of Its Kinetics and Substrate Specificity

**Figure 1:**
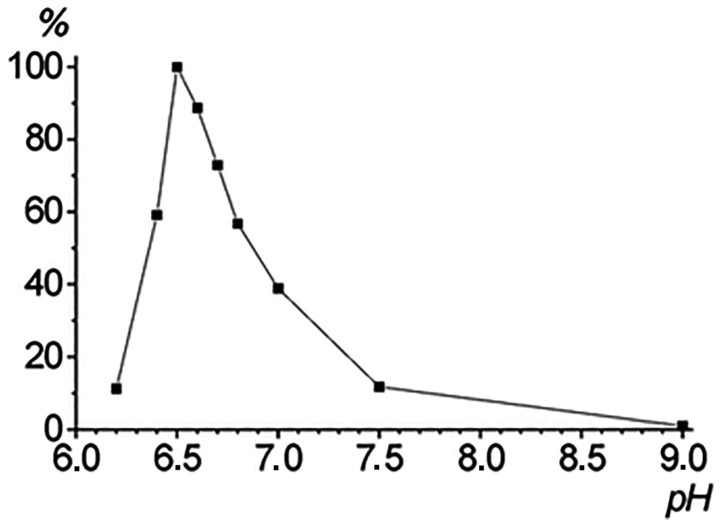
Effect of pH on activity of recombinant GmLOX2 with linoleic acid (140 μM) as substrate. Values represent the means of five replications.

The *GmLOX2* gene (GenBank GeneID: 547774) is localized on the 13th chromosome (43128326–43132622) and consists of 9 exons. Recombinant GmLOX2 has been obtained in a heterologous expression system using *E. coli* cells. The ORF of the *GmLOX2* gene, consisting of 2595 nucleotides and encoding an 865 amino acid polypeptide, has been subcloned into the pET-32 Ek/LIC vector, which allowed obtaining the target recombinant protein with His-tags at the N- and/or C-termini. His-tagged recombinant GmLOX2 was obtained and purified. The enzymatic activity was controlled using ultraviolet spectroscopy by the increase in fatty acid hydroperoxide absorbance at 234 nm. The pH optimum of the recombinant GmLOX2 was 6.5 (Figure 1). This result is in full agreement with previously published data for the affinity chromatography-purified GmLOX2 [22].

To evaluate the preference of recombinant GmLOX2 towards the ω3 or ω6 fatty acids, the kinetics of linoleic and α-linolenic acid conversions were studied. The *K_M_* values (Table 1) demonstrated that recombinant GmLOX2 affinity for α-linolenic acid was higher than for linoleic acid. The estimated turnover rate (*k_cat_*) of linoleic acid conversion was much higher than that of α-linolenic acid conversion (Table 1). As judged by specificity constant (*k_cat_*/*K_M_*) values, linoleic acid was the preferential substrate for recombinant GmLOX2. Thus, the kinetic data indicated that recombinant GmLOX2 utilized ω6 fatty acids more efficiently than ω3 fatty acids.

### 3.2. Products of C18 Fatty Acids Conversions by Recombinant GmLOX2

The reaction products of the recombinant GmLOX2 were analyzed in three different ways. Firstly, the methyl esters of NaBH_4_-reduced products were separated by NP-HPLC with ultraviolet diode array detection. Secondly, the Me esters/TMS derivatives (Me/TMS) of NaBH_4_-reduced products were also analyzed by GC-MS. The third analytic scheme included (1) NaBH_4_ reduction of products, (2) methylation with diazomethane, (3) hydrogenation of Me esters over PtO_2_, (4) trimethylsilylation, and (5) the GC-MS analyses of hydrogenated Me/TMS derivatives. The latter analytic scheme provided useful qualitative data for unambiguous product identification due to the backbone fragmentations and the oxy-TMS function.

**Figure 2:**
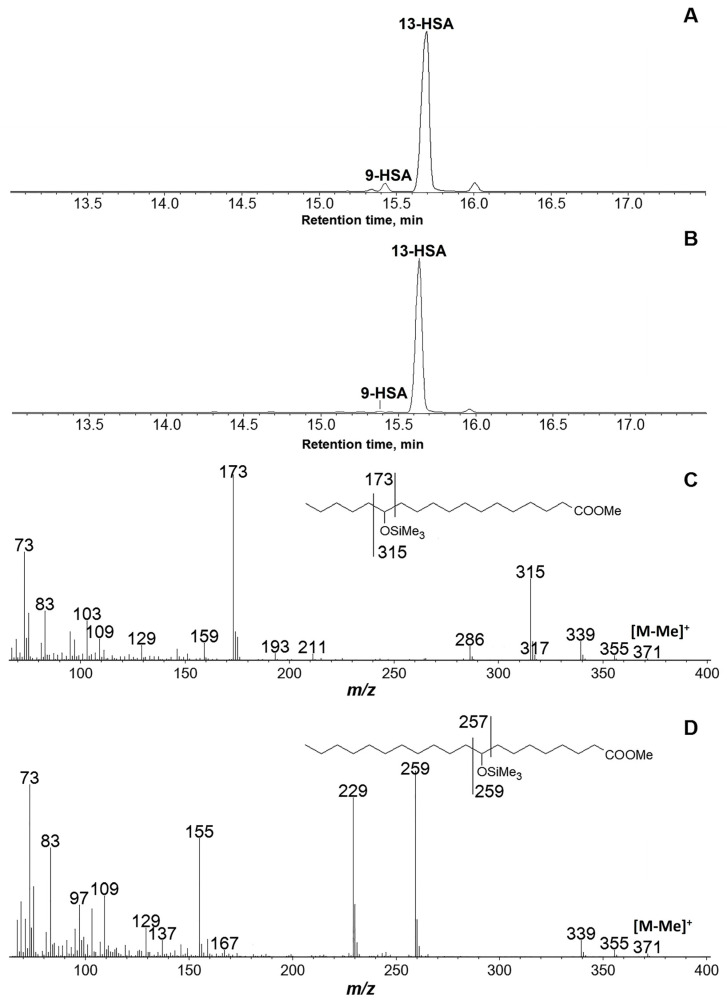
GC-MS analyses of products of conversion of linoleic (**A**) and α-linolenic (**B**) acids by the recombinant GmLOX2 after preliminary NaBH_4_ reduction and hydrogenation over PtO_2_; mass spectra of products (Me/TMS) of NaBH_4_ reduction and hydrogenation over PtO_2_ of 13-hydroperoxides (**C**) and 9-hydroperoxides (**D**) of linoleic and α-linolenic acids. 9-HSA, 9-hydroxystearic acid; 13-HSA, 13-hydroxystearic acid.

Incubation of the recombinant GmLOX2 with linoleic acid at optimal pH 6.5 yielded 13-HPOD as a predominant product, while 9-HPOD was a minority (Figure 2A). Separation of the NaBH_4_-reduced linoleic acid oxidation products (Me esters) by normal phase HPLC (NP-HPLC) with ultraviolet diode array detection revealed a 94:6 ratio of 13-HOD and 9-HOD Me esters (Table 2). The separated 13-HOD and 9-HOD (Me) samples were collected after the NP-HPLC separation and subjected to chiral phase HPLC (CP-HPLC) analyses. Enantiomeric separations showed that 13-HOD (Me) was composed predominantly of the (*S*)-enantiomer, while the minor product, 9-HOD (Me), was largely racemic (Table 2). When recombinant GmLOX2 was incubated with linoleic acid at pH 9.0, the oxidation proceeded slower than at the optimal pH value. The oxidation was also less regiospecific. The same 13-HPOD and 9-HPOD were formed at a ratio of 87:13. The enantiomeric compositions of both hydroperoxides were not altered compared to those formed at an optimal pH of 6.5.

Normally, the incubations are performed at a saturated oxygen concentration. Partly anaerobic incubations in the preliminary degassed buffer decreased the yield of 13-HPOD but did not induce the formation of anaerobic products like 13-oxo-9,11-tridecatrienoic acid (data not illustrated).

Oxidation of α-linolenic acid by the recombinant GmLOX2 afforded predominantly (92%) 13-HPOT, 6% 9-HPOT, and 2% 16-HPOT (Figure 2B). The 13-HPOT was composed of 96% 13(*S*)- and 4% 13(*R*)-enantiomers. The 9-HPOT and 16-HPOT were racemic.

The products were analyzed by SP- and CP-HPLC after reduction with sodium borohydride. The content of the products was determined by the peak area integration of the recorded UV data. The average values are presented.

### 3.3. Products of C20 Fatty Acids Conversions by Recombinant GmLOX2

**Figure 3:**
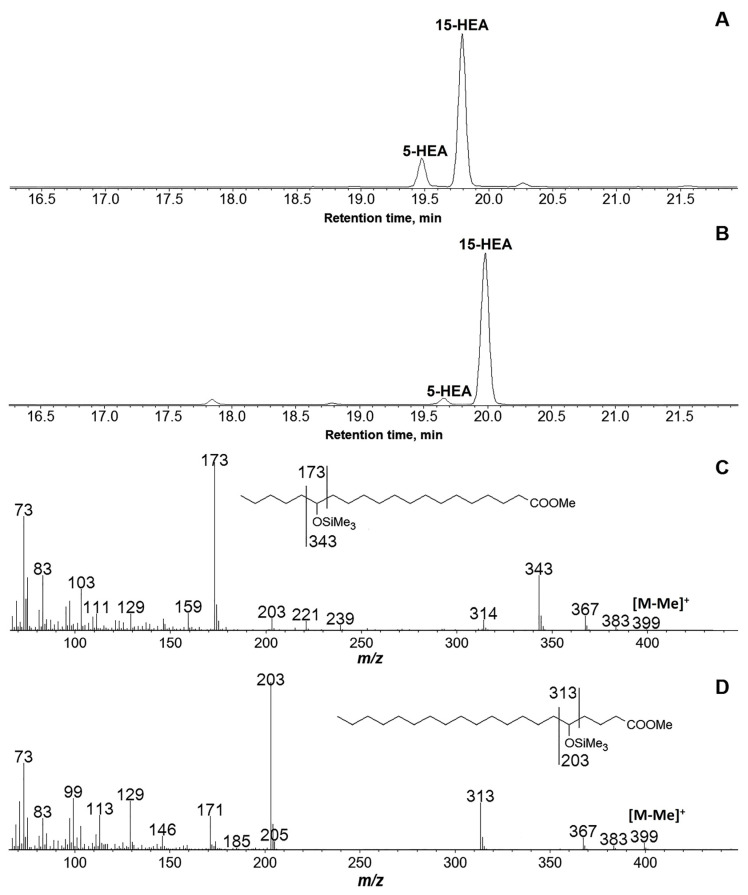
GC-MS analyses of products of conversion of arachidonic (**A**) and eicosapentaenoic (**B**) acids by the recombinant GmLOX2 after preliminary NaBH_4_ reduction and hydrogenation over PtO_2_; mass spectra of products (Me/TMS) of NaBH_4_ reduction and hydrogenation over PtO_2_ of 15-hydroperoxides (**C**) and 5-hydroperoxides (**D**) of arachidonic and eicosapentaenoic acids. 5-HEA, 5-hydroxyeicosanoic acid; 15-HEA, 15-hydroxyeicosanoic acid.

The GC-MS chromatograms of NaBH4-reduced and totally hydrogenated products (Me/TMS) of arachidonic and eicosapentaenoic acid oxidation by the recombinant GmLOX2 are illustrated in Figure 3A,B, respectively. As seen from these data, the predominant product of both arachidonic and eicosapentaenoic acid conversion was the compound mass spectrum, which (Figure 3C) possessed, inter alia, M^+^ at *m*/*z* 414 (0.01%), [M–Me]^+^ at *m*/*z* 399 (0.4%), [M–MeO]^+^ at *m*/*z* 383 (2%), [399–MeOH]^+^ at *m*/*z* 367 (9%), [M–Pe]^+^ at *m*/*z* 343 (0.4%), and [M–C1/C14]^+^ at *m*/*z* 173 (100%). The fragmentation patterns (Figure 3C, inset) matched those for 15-hydroxyeicosanoic acid (Me/TMS). Thus, the parent products of 20:4 and 20:5 oxidation by the recombinant GmLOX2 were 15-HPETE and 15-HPEPE, respectively.

The minor products of the conversion of arachidonic and eicosapentaenoic acids were 5-HPETE and 5-HPEPE, respectively. The mass spectra of the products of NaBH_4_ reduction and hydrogenation over PtO_2_ are presented in Figure 3D.

### 3.4. Products of Hexadecatrienoic Acid (C16) Conversion by the Recombinant GmLOX2

**Figure 4:**
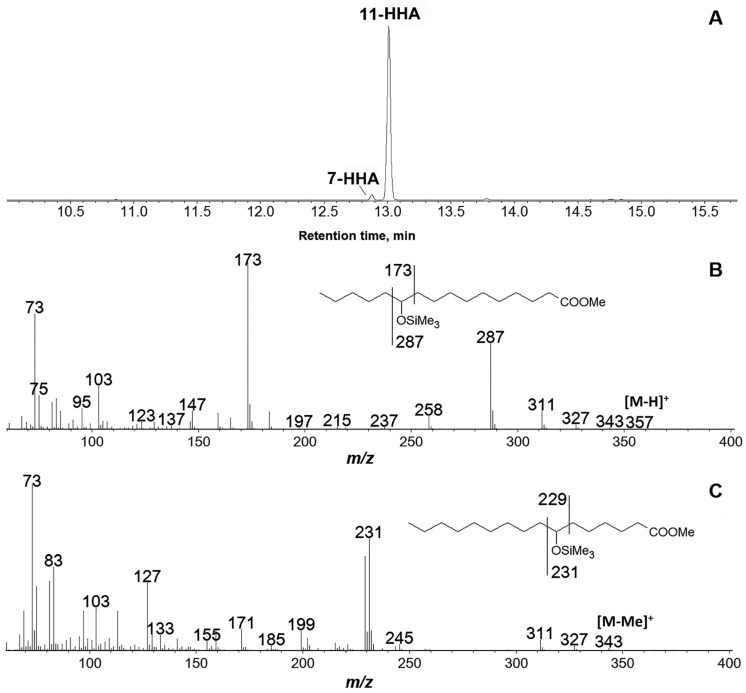
GC-MS analyses of products of conversion of hexadecatrienoic acid (**A**) by the recombinant GmLOX2 after preliminary NaBH_4_ reduction and hydrogenation over PtO_2_; mass spectra of products (Me/TMS) of NaBH_4_ reduction and hydrogenation over PtO_2_ of 11-hydroperoxide (**B**) and 7-hydroperoxide (**C**) of hexadecatrienoic acid. 7-HHA, 7-hydroxyhexadecanoic acid; 11-HHA, 11-hydroxyhexadecanoic acid.

As seen from Figure 4, the hexadecatrienoic acid (16:3) oxidation by the recombinant GmLOX2 resulted in a predominant product mass spectrometry, in which Me/TMS after the NaBH4 reduction and hydrogenation possessed [M–H]^+^ at *m*/*z* 357 (0.2%), [M–MeO]^+^ at *m*/*z* 327 (0.2%), [M–Me–MeOH]^+^ at *m*/*z* 311 (11%), [M–Pe]^+^ at *m*/*z* 287 (51%), [M–C11/C16 + TMS]^+^ at *m*/*z* 258 (8%), [M–C1/C10]^+^ at *m*/*z* 173 (100%), [CH_2_=O^+^–SiMe_3_] at *m*/*z* 103 (28%), and [SiMe_3_]^+^ at *m*/*z* 73 (73%). The fragmentation patterns (Figure 4B, inset) matched those for 11-hydroxyhexadecanoic acid (Me/TMS). These data indicated the structure of 11-hydroperoxy-7,10-hexadecadienoic acid for the original GmLOX2 product. The chiral phase HPLC analysis revealed the (*S*)-configuration of the asymmetric center at C11.

The minor product of the conversion of hexadecatrienoic acid was 7-HPHT. The mass spectrum of the products of NaBH_4_ reduction and hydrogenation over PtO_2_ is presented in Figure 4C.

## 4. Discussion

Early works on GmLOX2 have been performed with the enzyme isolated and purified from soybeans [16,17,18,19,20,21,22]. According to these data, GmLOX2 possessed uncertain regiospecificity, which varied from 75% of 9-HPOD [19] to 80% of 13-HPOD [16]. The results of current work unambiguously demonstrated the strict regiospecificity of the recombinant GmLOX2, which behaved as the ω6(*S*)-LOX towards several examined substrates, namely linoleic, α-linolenic, arachidonic, eicosapentaenoic, and hexadecatrienoic acids. The recombinant GmLOX2 converted these substrates into 13(*S*)-HPOD, 13(*S*)-HPOT, 15(*S*)-HPETE, 15(*S*)-HPEPE, and 11(*S*)-HPHT, respectively. These results indicated the similar specificity of GmLOX2 and GmLOX1. A major difference between these two isoenzymes is their pH dependence. In contrast to GmLOX1, which has an alkaline pH optimum, GmLOX2 possesses the highest activity at pH 6.5. Interestingly, no anaerobic cycle products like 13-oxo-9,11-tridecatrienoic acid were detected with GmLOX2. In contrast, GmLOX1 can produce 13-oxo-9,11-tridecadienoic acid as a major product under anaerobic conditions [29]. Presumably, GmLOX2 is less susceptible to oxygen concentration than GmLOX1.

**Figure 5:**
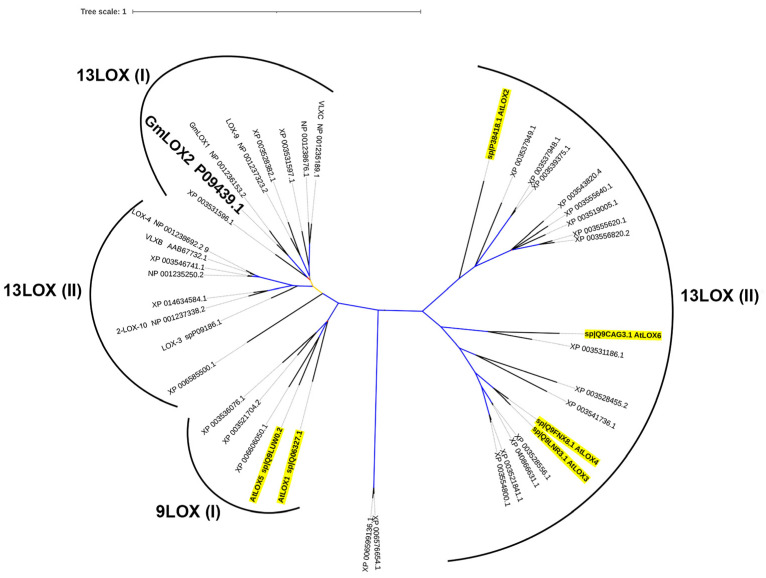
Phylogenetic analysis of *G. max* and *A. thaliana* LOXs. *G. max* LOXs: XP006585500.1, XP003521704.2, XP014634584.1, XP003521841.1, XP003528382.1, XP003536076.1, XP003546741.1, XP003554800.1, XP003531186.1, XP003531597.1, XP003528556.1, NP001237323.2 LOX9, AAB67732.1 VLXB, XP003556820.2, XP003537949.1, XP003519005.1, XP003555640.1, XP003555620.1, NP001237338.2 LOX-10, XP040866631.1, XP006599136.1, XP003537948.1, XP003539375.1, XP006606050.1, NP001238692.2 9(S)-specific LOX-4, XP003543820.4, NP001238676.1, XP006576654.1, XP003528455.2, NP001235189.1 VLXC, P09439.1 GmLOX2 (the object of the present research, highlighted in bold text and enlarged), XP003531596.1, P09186.1 LOX-3, NP001236153.2 13-LOX-1. *A. thaliana* LOXs (highlighted in bold and underlined text): Q06327.1 AtLOX1, P38418.1 AtLOX2, Q9LNR3.1 AtLOX3, Q9FNX8.1 AtLOX4, Q9LUW0.2 AtLOX5, Q9CAG3.1 AtLOX6. 9LOX(I)—9-LOXs type 1; 13LOX(I)—13-LOXs type 1; 13LOX(II)—13-lipoxygenases type 2. Blue branches—maximum bootstraps, yellow branches—medium bootstraps, red branches—minimum bootstraps.

**Figure 6:**
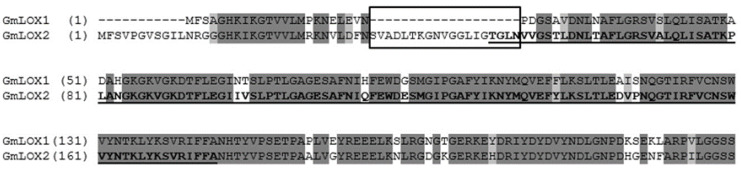
Alignment of partial *G. max* LOX1 and LOX2 sequences. The 19 amino acid deletion near the N-end in GmLOX1 is highlighted with the frame. The PLAT domain is defined according to the NCBI database (highlighted in bold and underlined text).

Similar catalytic specificities of GmLOX2 and GmLOX1 are not quite surprising, taking into account their close phylogenetic relationship. The mixed phylogenetic tree, composed of known soybean and *A. thaliana* LOX sequences, is presented in Figure 5. The branches were designated in accordance with the classification proposed earlier [2]. The GmLOX2, as well as the 13(*S*)-specific GmLOX1, are built in the neighborhood on the branch of 13-LOX type 2 (Figure 5). GmLOX2 possesses an 82.6% identity with GmLOX1. One of the main differences is the 19-amino acid deletion near the N-end of GmLOX1 (Figure 6). A comparison of all soybean LOXs shows that this deletion is a distinctive feature of GmLOX1 compared to other isoenzymes, including GmLOX2. One could propose that this difference between GmLOX2 and GmLOX1 may cause the distinct pH optima of these two isoenzymes. However, as found previously, the PLAT domain does not significantly affect the pH optima of LOXs [30].

**Figure 7:**
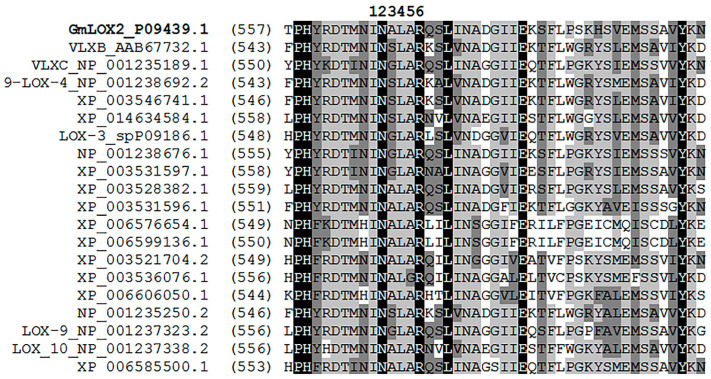
Multiple alignments of partial *G. max* LOX sequences annotated in the NCBI database as hypothetical 9-specific LOXs.

Only several G. max LOX genes have been cloned yet, namely three soybean seed genes encoding the L-1 (GmLOX1), L-2 (GmLOX2 [10]), and L-3 (GmLOX3), as well as five vegetative LOX genes encoding VLXA, VLXB, VLXC, VLXD, and VLXE [8,9]. The properties of corresponding recombinant proteins except GmLOX2 and GmLOX3 have been studied [8,9,10,11,12,13,14,15]. The properties of recombinant GmLOX2 were partly studied earlier [31]. The catalytic specificities of the great majority of soybean LOXs remain unexplored. The lack of data makes it difficult to properly predict the specificities of unstudied isoenzymes from their primary structures. For instance, GmLOX2 is annotated in the NCBI database as 9(*S*)-LOX. Moreover, several other soybean LOX isoenzymes (NP_001238692, XP_003546741, XP_006585500, etc.), which belong to the 13-LOX branch of the tree (Figure 5), are annotated in the NCBI database as 9(*S*)-LOXs. The inaccuracy of this attribution is confirmed by the similarity of the catalytically essential α-helix 11 (including the I-N-A/S/G-L/F-A-R motif [32] of GmLOX2 and other LOX sequences (Figure 7)). The I-N-A/S/G-L/F-A-R motif belongs to the U-shape pocket and is partly responsible for substrate positioning within the pocket [33]. Nevertheless, it is evidently problematic to predict the specificity of LOX from its primary structure. Only the preparation of recombinant enzymes allows one to reveal their properties unambiguously. However, the properties of LOXs are still often studied with protein preparations purified from plant tissues [34,35]. Even crystals for X-ray analyses were obtained using proteins purified from plant tissues [36].

Interest in soybean LOXs is due to their contribution to the quality of food raw materials and finished food products [37,38], including the rancidity of soybeans and the formation of volatile compounds, which produce an unpleasant odor of soybeans [28,39]. Soybean LOXs are used as model objects for studying the structure of LOXs [40,41,42,43,44,45,46,47] and for refining various techniques used, e.g., in industry [48,49].

Publications dated 2008 [50] and 2016 [51] revealed 19 and 36 LOX isoenzymes in the *G. max* genome, respectively. Further progress in sequencing and annotation provided new updates. Currently, 37 distinct expressed soybean LOX genes and 11 pseudogenes localized in 13 chromosomes are deposited in the NCBI Gene Database. Moreover, there are 12 more incomplete LOX sequences. Thus, it is likely that the number of *G. max* LOX isoenzymes can be increased in the course of forthcoming sequencing and annotation. So, the Glyma2.0 Gene Model in the SoyBase database https://www.soybase.org/ (accessed on 30 June 2023) includes a total of 55 genes (or pseudogenes) encoding LOX isoenzymes and is localized in 14 chromosomes. The great majority of GmLOX isoenzymes have not been studied yet. The functional and physiological significance of this diversity awaits explanation.

## Figures and Tables

**Table 1:** Kinetic parameters and substrate specificity of recombinant GmLOX2.

Substrate	*k_cat_* (s^−1^)	*K_M_* (μM)	*k_cat_*/*K_M_* (μM^−1^∙s^−1^)	Specificity, %
18:2, linoleic acid	61.9 ± 2.8	10.6 ± 1.2	5.8	100
18:3, α-linolenic acid	66.6 ± 3.8	20.8 ± 1.5	3.2	55.17

**Table 2:** Regio- and stereospecificity of linoleic acid oxygenation by the recombinant GmLOX2.

pH	13-HPOD/9-HPOD	13*S*-/13*R*-HPOD	9*S*-/9*R*-HPOD
6.5	94:6	95:5	52:48
9.0	87:13	95:5	52:48

## Data Availability

Not applicable.
